# Protective Mechanism of Sulforaphane on Cadmium-Induced Sertoli Cell Injury in Mice Testis via Nrf2/ARE Signaling Pathway

**DOI:** 10.3390/molecules23071774

**Published:** 2018-07-19

**Authors:** Shu-Hua Yang, Li-Hui Yu, Lin Li, Yang Guo, Yi Zhang, Miao Long, Peng Li, Jian-Bin He

**Affiliations:** Key Laboratory of Zoonosis of Liaoning Province, College of Animal Science & Veterinary Medicine, Shenyang Agricultural University, Shenyang 110866, China; yangshuhua0001@syau.edu.cn (S.-H.Y.); yuyang75060@163.com (L.-H.Y.); lilin619619@163.com (L.L.); jessekuo@163.com (Y.G.); sihuo12345@sohu.com (Y.Z.)

**Keywords:** sulforaphane, Cd, TM4 cell, apoptosis, oxidative damage, Nrf2/ARE signaling pathway

## Abstract

The present study evaluated the mechanism underlying the protective effect of sulforaphane (SFN) on cadmium (Cd)-induced Sertoli cell (TM4 cells) injury in mice. The apoptosis rate of cells in each group was detected by flow cytometry. It was determined the effect of SFN on the expression of downstream molecular targets of Nrf2/ARE axis and on the lipid peroxide content. The related genes involved in the nuclear factor E2-related factor 2(Nrf2)/antioxidant response element (ARE) signaling pathway were evaluated by RT-PCR; for example, the mRNA expression levels of Nrf2, heme oxygenase-1 (HO-1), glutathione peroxidase (GSH-Px), quinone oxidoreductase 1 (NQO1), and γ-glutamylcysteine synthetase (γ-GCS), while the protein expression levels were assessed by Western blot. Our results showed that the mRNA and protein expression levels of Nrf2, HO-1, NQO1, GSH-Px, and γ-GCS were increased in various degree when the Sertoli cells were to added different concentrations of SFN. Our results also showed that SFN reduced the apoptosis rate, increased the activity of T-SOD, inhibited the increase of the MDA content caused by Cd. Meanwhile, SFN could increase the mRNA and protein expression levels of Nrf2, HO-1 and NQO1 and reduced the mRNA and protein expression levels of GSH-Px and γ-GCS caused by Cd in Sertoli cells (*p* < 0.01). Taken together, SFN could improve the antioxidant capacity of Sertoli cells, and exert a protective effect on the oxidative damage and apoptosis of Cd-induced Sertoli cells through the activation of Nrf2/ARE signal transduction pathway.

## 1. Introduction

Cadmium (Cd) is a well-known environmental pollutant and a major industrial raw material, wherein it is primarily used for manufacturing batteries, metal electroplating, pigments, plastic cement, and alloy. Typically, the organisms are poisoned by ingesting Cd-contaminated food and drinking water, while humans could be poisoned when they are engaged in the processing and use of batteries and pigments; also, some poisoning could be attributed to smoking [1,2]. Cd poisoning could lead to liver and kidney dysfunction, bone injury, testicular injury, and respiratory and nervous system disorders [3,4]. Of these, the reproductive toxicity is one of the main reactions of Cd poisoning in male animals. In Cd-exposed rats, the ratio of the testis to body weight decreased significantly, Cd accumulated in the testicular tissues, the levels of lipid peroxides and protein carbonyl increased significantly, and the antioxidant defense ability of testicular tissues decreased [5]. On the other hand, in the Cd treatment group, the average diameter of the seminiferous tubule of the testis, the average of the score of testicular biopsy, and the level of serum testosterone decreased significantly, and testicular cells succumbed to apoptosis [6]. Furthermore, Cd increased the level of lipid peroxidation and the number of abnormal sperm in rat testis, reduced the level of plasma testosterone, testicular steroid synthesis as well as the number and motility of sperms in the epididymis [7], which caused a severe weight loss of rats, testicular atrophy, necrosis, fibrosis, calcification, and significant peripheral lymphocyte infiltration [8]. Another study showed that the final body weight, relative testicular weight, the weight of epididymis, the weight of the epididymis, the weight of semen, the weight of prostate of Cd-poisoned rats, the activity of antioxidant enzymes in the testicular tissues, and the androgen receptors in rat testis decreased, while the protein expression of caspase-3 increased [9]. Combined with previous studies, we found that antioxidants and antioxidant defense systems played critical roles in protecting the male reproductive system from Cd-induced oxidative damage. Thus, the major negative impacts of Cd on animal reproduction and human health has become a global concern. However, an effective treatment for Cd poisoning in animals and humans was lacking.

In recent years, some natural antioxidants have been used to combat Cd toxicity, while some studies reported about reducing the reproductive toxicity. For example, quercetin [10], curcumin [11], proanthocyanidins [12], *Crocus sativus* L. [13], grape juice concentrate [14], and *Lycium triterpenes* [15] could reduce the oxidative stress and reproductive toxicity caused by Cd poisoning. Sulforaphane (SFN) is an isothiocyanate, mainly from broccoli, radish, leaf mustard, horseradish, *Brassica oleracea* vat. Botrytis, *Oenanthe benghalensis* Benth et al. Hook and other *Brassicaceae* burnetts; the maximum SFN content was found in broccoli. SFN is recognized worldwide as an anti-cancer botanical, antioxidant and as the finest natural active substance for scavenging free radicals [16,17]. SFN also activates Nrf2, which in turn, regulates the expression of phase II detoxification enzyme [18]; in addition, it detoxifies and exerts a protective effect on the damage caused by environmental chemical toxicants. Owing to the wide range of source, SFN has broad application prospects for the protection of human and animal health. A previous study showed that SFN pretreatment could antagonize the cytotoxicity and neurotoxicity of methylmercury, as well as reduce the accumulation of methylmercury in the brain, cerebellum, and liver of the rat [19]. After SFN pretreatment in rats, the antioxidant ability of kidney tissues was improved, and an antagonistic effect on the Cd-induced kidney damage was detected [20]. In vitro studies showed that at 24 h after SFN pretreatment, the accumulation of arsenic in hepatocytes decreased, and liver cell viability increased significantly [21]. When the cells were cultured in the presence of SFN or Cd alone or the combination of both, the SFN was found to decrease the cytotoxic effect of Cd-induced lymphocytes and monocytes [22]. Furthermore, it was used to infuse the rats that were polluted with Cd, Moreover, SFN exhibited a robust antioxidant effect, the concentration of serum testosterone and daily sperm production in rats increased significantly, and the Cd-induced reproductive toxicity was antagonized [23]. Our previous study also found that SFN could significantly reduce the Cd-induced oxidative damage of testicular tissues and the reproductive toxicity [24]. However, the effect of SFN on Cd-induced oxidative cellular damage and the underlying protective mechanism are yet to be elucidated.

Therefore, the present study investigated the protective effect of SFN on Cd-induced oxidative cellular damage in rat testis and explore its mechanism. The results provided a theoretical basis for the prevention and control of the cytotoxicity of Cd, as well as, the application of SFN in production practice.

## 2. Experimental Design and Treatment

### 2.1. Effects of Different Concentrations of Cd on Cell Viability

2 × 10^5^ cells/mL TM4 were cultured in 96-well plate (300 μL in each well) and incubated at 37 °C, 5% CO_2_ for 6 h. After adherence, 20 µL cadmium at 0, 0.0975, 0.195, 0.39, 0.78, 1.56, 3.125, 6.25, 12.5, 25, 50, 75, 100, 125, 150, 200, and 400 μmol/L were added to the culture plate, four wells each, respectively, followed by incubation for 24 h. Subsequently, the supernatant was discarded, MTT reagent (10 μL) and serum-containing culture medium (90 μL) added, incubated for 4 h, the supernatant was discarded, and Formazan solution (110 μL) was added to each culture plate. The absorbance was measured at 490 nm, and the survival cells calculated as follows: Relative survival = (Absorbance value of experimental group/absorbance value of control group) × 100%; half maximal inhibitory concentration (IC50) was used as a reference for the subsequent experiments. IC50 was calculated using the SPSS17.0 software (IBM, Almon, NY, USA).

### 2.2. Effect of SFN on Cell Viability

The cell viability was detected as described above. The concentrations of SFN were 0, 2.5, 5, 10, 20, 40, 80, and 160 μmol/L, respectively.

### 2.3. Effects of SFN and Cd on Cell Viability

The concentration of Cd in the Cd group was 12.5 μmol/mL, while that of SFN in the SFN group was 0.625, 2.5, and 10 μmol/L, respectively. The cell viability was detected as described above. 

### 2.4. Effects of SFN and Cd on the Activity of LDH in Cells

The concentration of Cd in the Cd group was 12.5 μmol/L, while that of SFN in the SFN group was 0.625, 2.5, and 10 μmol/L, respectively. Cd and SFN group. The activity of LDH was determined according to the instructions of the 2,4-dinitrobenzene colorimetric assay kit (Nanjing Jiancheng Bioengineering Institute, Nanjing, China).

### 2.5. Apoptosis Rate Assessed by Flow Cytometry

The grouping was conducted according to the operation method described in the section 1.3 of the study. At 24 h post-incubation, the cells were trypsinized and collected by centrifugation at 1000 rpm for 5 min. Then, the cells were suspended in Annexin V binding buffer (100 μL) and FITC Annexin V (5 μL) with propidium iodide solution (10 μL) in the dark for 15 min at room temperature (The flow-type FITC Annexin V Apoptosis Detection Kit, Beijing Dakewe, China). Finally, the cells were analyzed by flow cytometry (Becton, Dickinson and Company, Franklin Lakes, NJ, USA) in 400 µL Annexin V binding.

### 2.6. Detection of Oxidation Damage

The level of oxidative stress in TM4 cells was based on the detection of the MDA content and T-SOD enzyme activity using a commercial assay kit.

### 2.7. Detection of mRNA Expression 

TRIzol method was used to extract total RNA from TM4 cells, and agarose gel electrophoresis was used to detect whether RNA was degraded or mixed with impurities. Absorbance value was determined. When the OD260/OD280 ratio was between 1.8 and 2.1, the purity of RNA was high. Reverse transcription kit was used for RNA reverse transcription. The primer 5.0 software (IBM, Almon, NY, USA) was used to design primers, and their specificities were verified by Oligo7. The designed primers were submitted to the Shanghai Bioengineering Co., Ltd. (Shanghai, China) for the completion of synthesis. The primer sequences are shown in Table 1. Quantitative real-time RT-PCR was performed by a fluorescent quantitative gene amplification instrument (IQ5, ABI, Waltham, MA, USA). Reaction systems included the following parts: Total reaction system 20 μL (10 μL of SYBR Premix Ex Taq, 0.5 μL of forward primer and reverse primer, respectively, 1 μL of cDNA and 8 μL ofdH_2_O). The operations were conducted on the ice according to the instructions of kit. 2^−ΔΔCT^ method was used for real-time PCR data analysis.

### 2.8. Western Blot

Total protein was extracted using RIPA lysis buffer. An equivalent of 15–30 μg protein extract was resolved by SDS-PAGE and transferred to nitrocellulose membrane that was blocked with BSA for 2 h. Then, the membrane was probed with primary antibodies against Nrf2, GSH-Px, HO-1, γ-GCS, NQO1, and β-actin overnight at 4 °C (Santa Fe, NM, USA) and secondary antibody at room temperature (Santa Fe, NM, USA). Image analysis system was used for the quantitative analysis of target protein expression (Beyotime Institute of Biotechnology, Shanghai, China). 

### 2.9. Statistical Analysis

The SPSS17.0 software was used for statistical analysis. The differences among the groups were analyzed by ANOVA (IBM, Almon, NY, USA). (Discrete analysis), and LSD was used for multiple comparisons. The results were expressed as mean ± standard deviation. *p* < 0.05 was considered as difference, and *p* < 0.01 was considered as significant difference.

## 3. Results

### 3.1. Survival Rate of Cd-Induced TM4 Cells

**Figure 1:**
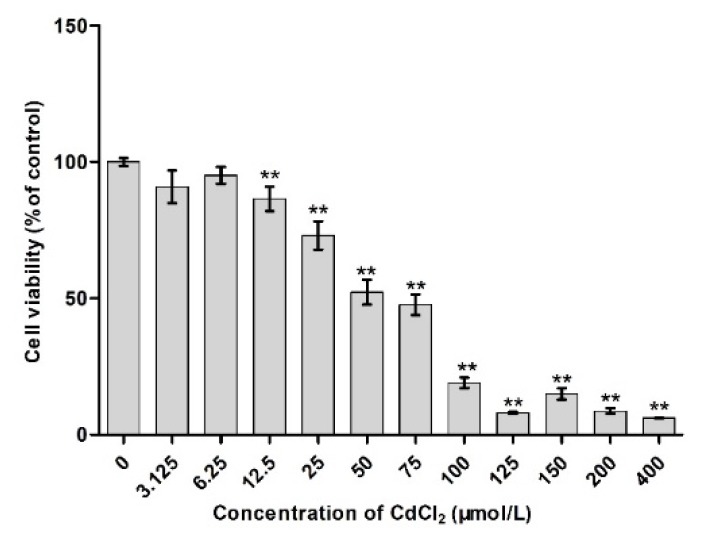
Effect of Cd on the activity of TM4 cells. ** *p* < 0.01 as compared to the control group.

As shown in Figure 1, compared to the control group, the survival rate of TM4 cells did not change when the concentration of Cd was 0.0975–6.25 μmol/L (*p* > 0.05). However, the survival rate of TM4 cells decreased significantly when the concentration of Cd was 12.5 μmol/L (*p* < 0.01) in a concentration-effect manner. In this study, the half-maximal inhibitory concentration of Cd to TM4 cells was 56.082 μmol/L.

### 3.2. Effect of SFN on the Survival Rate of TM4 Cells

**Figure 2:**
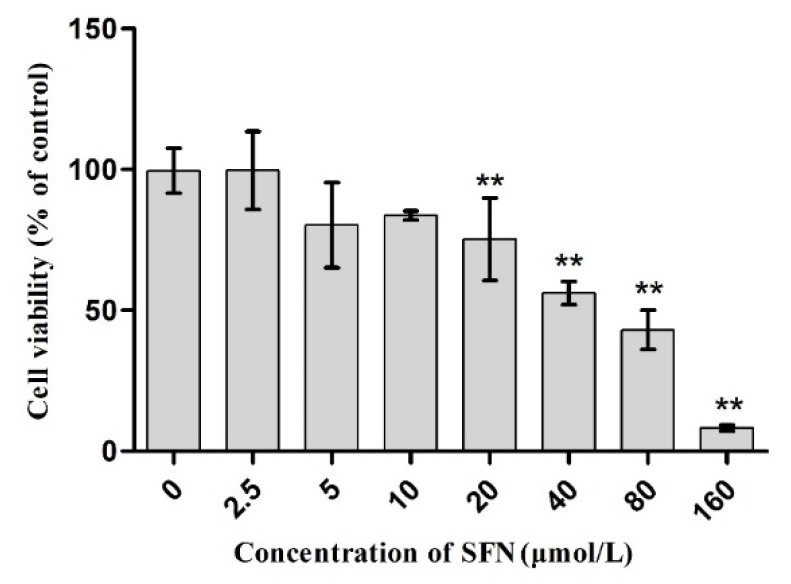
Effect of SFN on the activity of TM4 cells. ** *p* < 0.01 as compared to the control group.

As shown in Figure 2, compared to the control group, the survival rate of TM4 cells did not decrease significantly when the concentration of SFN was between 0 and 10 μmol/L (*p* > 0.05); however, the survival rate of TM4 cells decreased significantly with the increase in SFN concentration that was >20 μmol/L (*p* < 0.01).

### 3.3. Effects of Different Concentrations of SFN on the Viability of Cd-Exposed TM4 Cells

**Figure 3:**
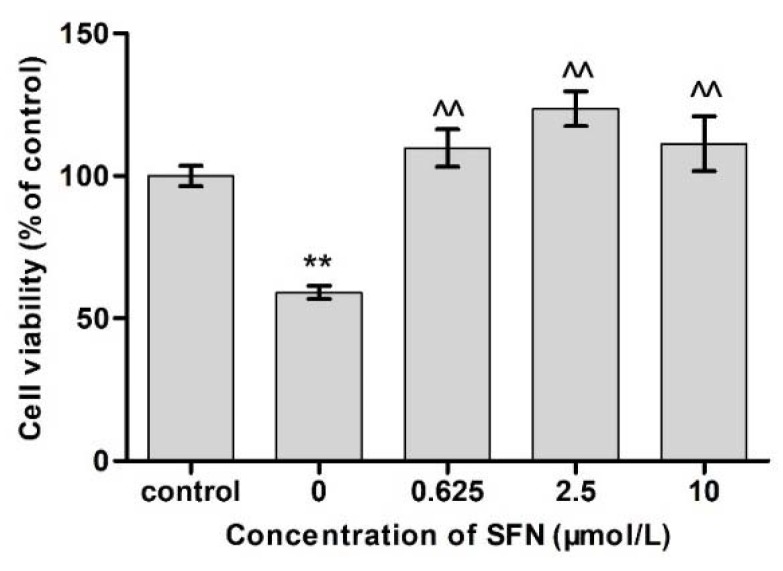
Effects of SFN at different concentrations on the viability of Cd-exposed TM4 cells (12.5 μmol/L). ** *p* < 0.01 as compared to the control group, ^^ *p* < 0.01 as compared to the Cd group.

As shown in Figure 3, the survival rates of TM4 cells in the Cd + SFN0.625 Cd + SFN2.5 and Cd + SFN10 groups were significantly higher than that of the Cd + SFN0 group (*p* < 0.01), and Cd group was significantly lower than that of the control group.

### 3.4. Detection of TM4 Cell Activity by LDH

**Figure 4:**
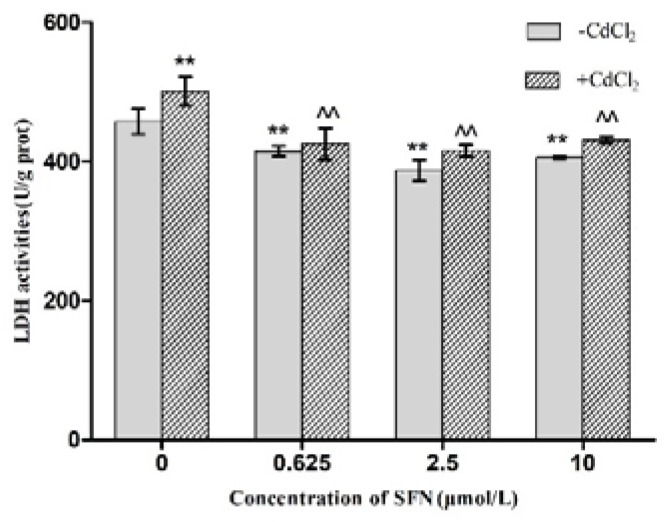
Effect of SFN on the LDH activity of Cd-exposed TM4 cells * *p* < 0.05 as compared to the control group, ** *p* < 0.01 as compared to the control group, ^^ *p* < 0.01 as compared to the Cd group.

In order to determine the putative effect of SFN on reducing Cd induced cytotoxicity, LDH activity was analyzed. As shown in Figure 4, the activity of LDH in TM4 cells was detected, and the Cd group was found to be significantly higher than that of the control group (*p* < 0.01). Compared to the control group, the LDH activities in the SFN0.625 group, SFN2.5 group, and SFN10 group decreased significantly (*p* < 0.01). Furthermore, LDH test showed that the LDH activities of Cd + SFN0.625, Cd + SFN2.5, and Cd + SFN10 group were lower as compared to the Cd group (*p* < 0.01).

### 3.5. Effect of Cd-Induced SFN on the Apoptosis of TM4 Cells

**Figure 5:**
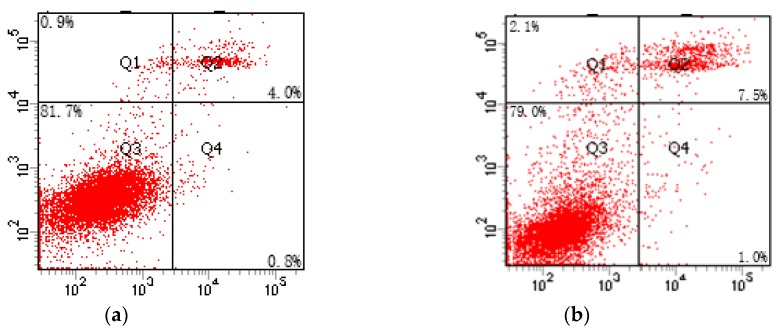

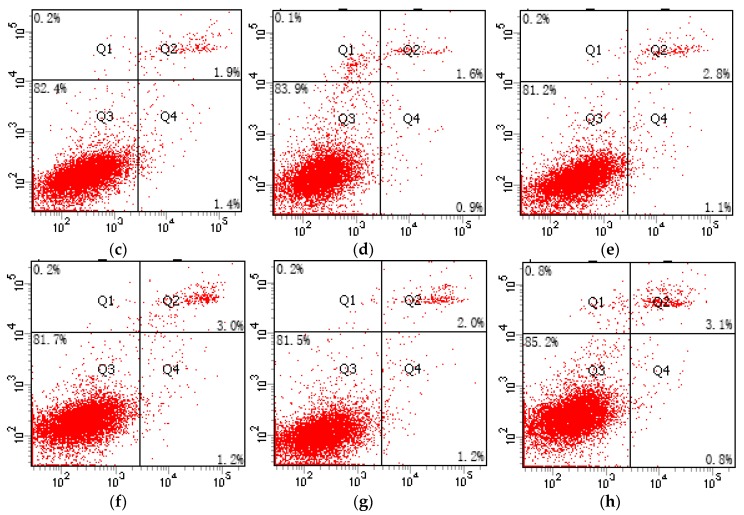
Effect of SFN on the apoptosis of Cd-induced TM4 cells. (**a**) Control group; (**b**) Cd group; (**c**) SFN0.625 group; (**d**) SFN2.5 group; (**e**) SFN10 group; (**f**) Cd + SFN0.625 group; (**g**) Cd + SFN2.5 group; (**h**) Cd + SFN10 group.

As shown in Table 2 and Figure 5, the apoptosis rate of TM4 cells in the Cd experimental group increased significantly as compared to the control group (*p* < 0.01), while the apoptosis rates of TM4 cells in the SFN2.5, SFN5, and SFN10 groups decreased significantly (*p* < 0.01). In addition, compared to the Cd group, the apoptosis rates in the Cd + SFN2.5, Cd + SFN5, and Cd + SFN10 groups decreased significantly (*p* < 0.01). When the concentration of SFN was 5 μmol/L, the protective effect against Cd-induced apoptosis was maximal.

### 3.6. Effects of SFN on Factors Related to Oxidative Stress in Cd-Induced TM4 Cells

**Figure 6:**
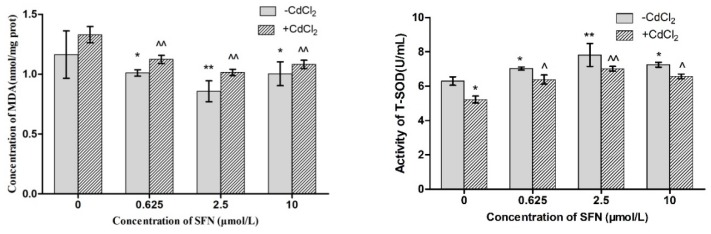
Effects of SFN on the MDA content and T-SOD activity in Cd-exposed TM4 cells * *p* < 0.05 as compared to the control group, ** *p* < 0.01 as compared to the control group, ^ *p* < 0.05 as compared to the Cd group, ^^ *p* < 0.01 as compared to the Cd group.

MDA (malondialdehyde) content was measured to determine lipid peroxidation levels. As shown in Figure 6, the activity of T-SOD in the Cd group decreased as compared to the control group, (*p* < 0.05), while the activities of T-SOD in the Cd + SFN0.625, Cd + SFN2.5, and Cd + SFN 10 group increased as compared to the Cd group (*p* < 0.05, *p* < 0.01). Furthermore, compared to the control group, the MDA content in the Cd group increased, while the content in the SFN group was lower than that of the control group (*p* < 0.05, *p* < 0.01). Compared to the Cd group, the MDA content in the Cd + SFN0.625, Cd + SFN2.5, and Cd + SFN10 groups decreased significantly (*p* < 0.01).

### 3.7. Effects of SFN and Cd on the mRNA and Relative Protein Expressions of Nrf2, HO-1, NQO1, GSH-Px, and γ-GCS in TM4 Cells

**Figure 7:**
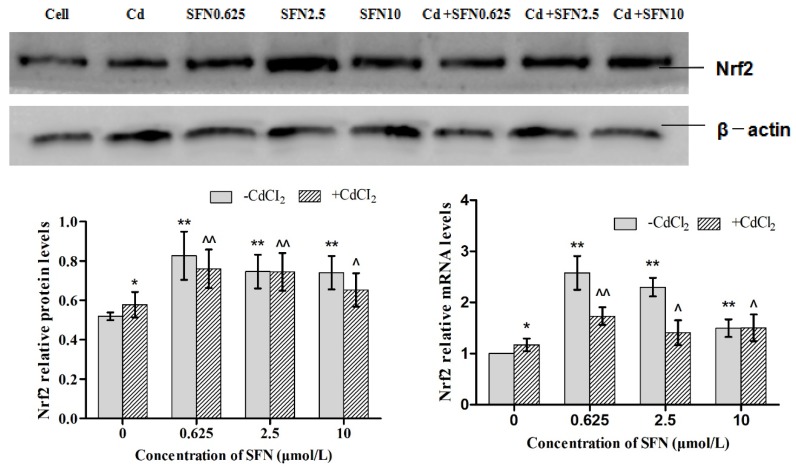
Effects of SFN on Cd induced the relative mRNA and protein expression of Nrf2 of the TM4 cells. * *p* < 0.05 as compared to the control group, ** *p* < 0.01 as compared to the control group, ^ *p* < 0.05 as compared to the Cd group, ^^ *p* < 0.01 as compared to the Cd group.

**Figure 8:**
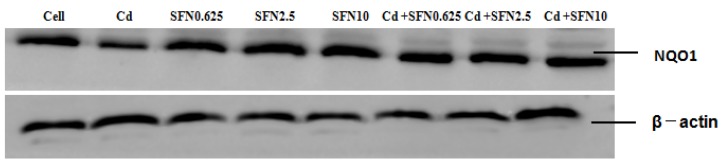

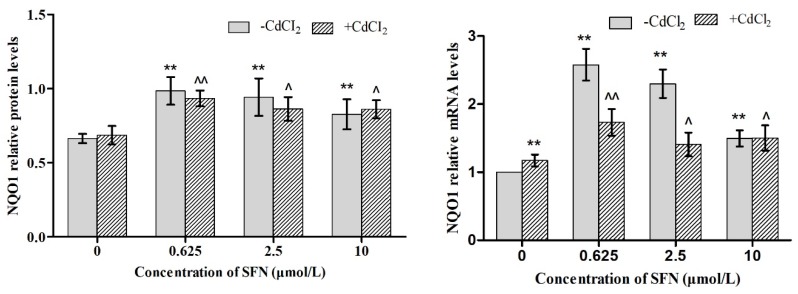
Effects of SFN on Cd induced the relative mRNA and protein expression of NQO1 of the TM4 cells. ** *p* < 0.01 as compared to the control group, ^ *p* < 0.05 as compared to the Cd group, ^^ *p* < 0.01 as compared to the Cd group.

**Figure 9:**
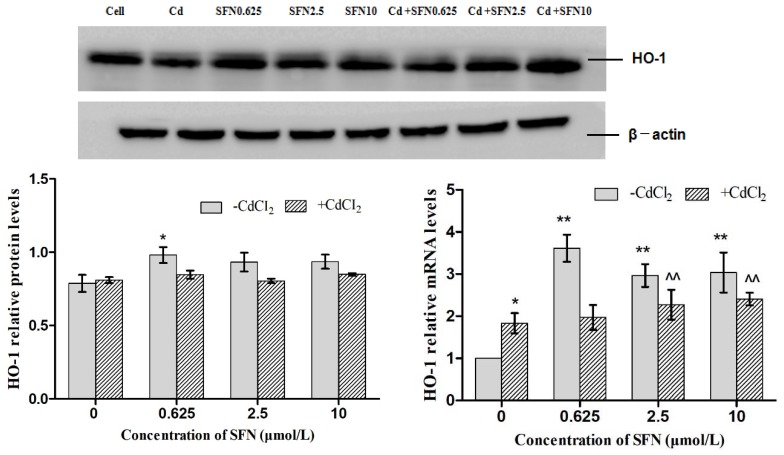
Effects of SFN on Cd induced the relative mRNA and protein expression of HO-1 of the TM4 cells. * *p* < 0.05 as compared to the control group, ** *p* < 0.01 as compared to the control group, ^^ *p* < 0.01 as compared to the Cd group.

**Figure 10:**
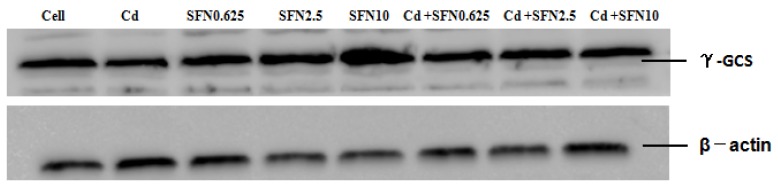

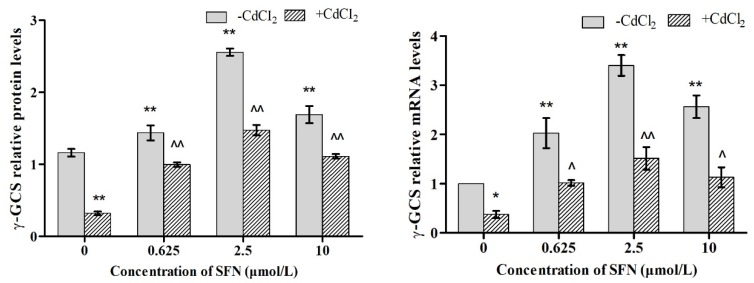
Effects of SFN on Cd induced the relative mRNA and protein expression of γ-GCS of the TM4 cells. * *p* < 0.05 as compared to the control group, ** *p* < 0.01 as compared to the control group, ^ *p* < 0.05 as compared to the Cd group, ^^ *p* < 0.01 as compared to the Cd group.

**Figure 11:**
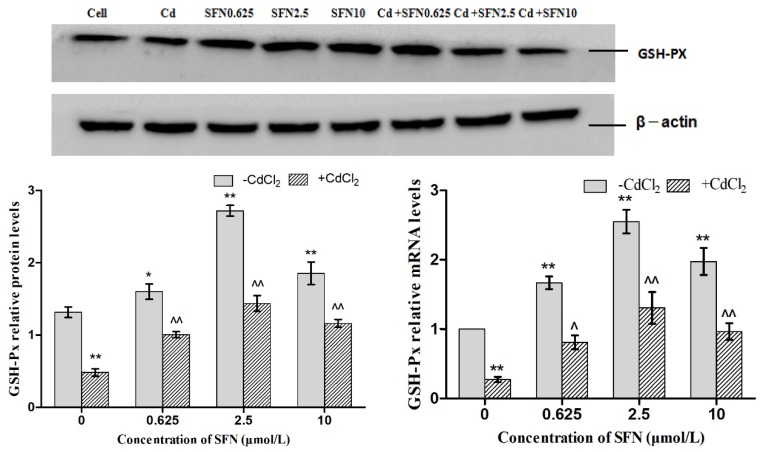
Effects of SFN on Cd induced the relative mRNA and protein expression of GSH-Px of the TM4 cells. * *p* < 0.05 as compared to the control group, ** *p* < 0.01 as compared to the control group, ^ *p* < 0.05 as compared to the Cd group, ^^ *p* < 0.01 as compared to the Cd group.

As shown in Figure 7, Figure 8, Figure 9, Figure 10 and Figure 11, the mRNA levels of Nrf2, HO-1 and NQO1 in the Cd group increased significantly (*p* < 0.01, *p* < 0.05), the relative protein expressions also increased but not significantly as compared to the control group. The mRNA and protein expressions of *GSH-Px* and γ-GCS decreased significantly (*p* < 0.01, *p* < 0.05) as compared to the control group. Compared to the control group, the mRNA levels of *Nrf2, NQO1, γ-GCS, GSH-Px, HO-1* and the protein expressions of *Nrf2*, *NQO1, γ-GCS* and *GSH-Px* in the SFN0.625, SFN2.5, and SFN10 groups increased significantly (*p* < 0.01, *p* < 0.05). The protein expressions of HO-1 in SFN0.625 group increased significantly as compared to the control group (*p* < 0.05).

The mRNA and protein expressions of *Nrf2, NQO1, γ-GCS* and *GSH-Px* in the Cd+SFN0.625, Cd + SFN2.5 and Cd + SFN10 groups increased significantly (*p* < 0.05, *p* < 0.01) as compared to the Cd group. The mRNA expressions of *HO-1* in the Cd + SFN2.5 and Cd + SFN10 group increased significantly (*p* < 0.01), while no significant differences of *HO-1* protein expressions were noted in the Cd + SFN0.625, Cd + SFN2.5 and Cd + SFN10 as compared to the Cd group. 

## 4. Discussion

Cadmium (Cd) is a well-known heavy metal that could cause environmental pollution, as well as, human and animal health problems. Cd has multiple-organ and multi-system toxicity; for example kidney toxicity, hepatotoxicity, heart toxicity, bone toxicity, reproductive toxicity, neurotoxicity, immunotoxicity, genotoxicity, and carcinogenesis [25]. Reportedly, several safety and efficacy issues were noted while using chelating agents for the treatment of Cd toxicity [26]. Therefore, developing an effective agent for the treatment of Cd poisoning was essential. SFN was abundant in cruciferous plants with the effects of detoxification, antioxidation, inhibiting inflammation, and immunoregulation [27,28,29]; it was considered as an active foodborne component with high value. Our previous study showed that SFN alleviated the testicular toxicity induced by Cd in rats, increase the total number of sperm in the epididymis, and reduce the ratio of abnormal sperm [24]. However, the specific mechanism was not yet clarified.

In this study, TM4 cells, the main constituent of blood-testis barrier, were selected to investigate the mechanism underlying the effect of SFN on the toxicity of Cd. The results showed that the half maximal inhibitory concentration of Cd on TM4 cells was 56.082 μmol/L; when the concentration of Cd was >12.5 μmol/L, the proliferation of TM4 cells was significantly inhibited (Figure 1) and the cell activity decreased. This phenomenon might be attributed to the Cd-induced apoptosis (Table 2, Figure 5). The antioxidant effect of SFN was noted, when its concentration was >20 μmol/L, and the survival rate of TM4 cells decreased significantly with increasing SFN concentration, i.e., a high concentration of SFN inhibited cell proliferation (Figure 2). This might be attributed to SFN as a chemical substance of isothiocyanate, which could produce cytotoxicity in the case of high SFN concentration in the culture medium. LDH was an intracellular enzyme; when the cells were broken or damaged, LDH is an intracellular enzyme which is released when cells are broken or damaged. Thus, to analyze the protective effect of SFN against Cd, LDH activity was measured [30]. We found that the activity of LDH in the culture medium of the Cd group increased significantly (*p* < 0.05); after the cells were cultured in the combination of Cd and SFN, the activity of LDH in the supernatant was significantly lower than that of the group treated with Cd only (*p* < 0.01) (Figure 4). The current results indicated that SFN could effectively prevent Cd-induced cell membrane damage and reduce LDH escape from the cells.

Previous studies have shown that Cd could induce apoptosis under in vitro culture conditions, such as the primary cell apoptosis of proximal renal tubules in rats [31], the apoptosis of human embryonic gonadal germ cells [32], the apoptosis of primary osteoblasts [33], and the apoptosis of human peripheral lymphocytes and monocytes [34]. Under the current experimental conditions, Cd induced the apoptosis of TM4 cells in rats, while SFN could reduce the toxicity of Cd and reduce the apoptosis of TM4 cells (Table 2, Figure 5). The protective effect of SFN on apoptosis was similar to that reported in the study by Nouf et al. [34,35]. Thus, we speculated that Cd might disrupt the blood-testis barrier by damaging the TM4 cells, further affecting the spermatogenic function in male mice.

Herein, we found that Cd could reduce the antioxidant defense ability of TM4 cells; also, the activity of T-SOD was decreased, and MDA content was increased (Figure 6). Next, we speculated that oxidative stress might be the cause of apoptosis of Cd-induced TM4 cells. Therefore, SFN was simultaneously added in the culture medium-containing Cd to investigate the antioxidant effect of SFN on TM4 cells. The results showed that after adding SFN, the antioxidant effect (T-SOD activity) of TM4 cells increased, and the number of apoptotic cells decreased as compared to those of the group that was treated with Cd only (Figure 6), suggesting satisfactory anti-oxidant and anti-apoptotic effects of SFN that could protect TM4 cells and reduce the Cd-induced toxicity.

Nrf2 is a vital transcription factor in oxidative stress that promotes cell survival and maintains cell redox homeostasis by regulating the expressions of phase II detoxification enzymes and antioxidant enzymes in cells [29,36]. The Nrf2/ARE signaling pathway is a crucial antioxidant pathway that is widely distributed in the histiocytes of the whole body [37,38,39]. Therefore, in order to investigate whether SFN could inhibit the damage of Cd-induced TM4 cells by activating the Nrf2/ARE signaling pathway, the changes in the expressions of some related genes in the Nrf2/ARE signaling pathway were detected in the present study. The results showed that the mRNA and protein expression of Nrf2 in the cells of Cd treatment group increased significantly, and that of HO-1, NQO1 downstream to the Nrf2 signaling pathway increased in varying degrees, and the mRNA (Figure 7, Figure 8 and Figure 9) and protein expression of γ-GCS, GSH-Px in the cells of Cd treatment group decreased significantly (Figure 10 and Figure 11). These results confirmed that exogenous Cd could activate the Nrf2/ARE signaling pathway, and increase the intrinsic antioxidative capacity of TM4 cells. The activities of these antioxidant enzymes would decrease when the expressions of protein of γ-GCS and GSH-Px were inhibited by Cd. The reason might be that cadmium had high affinity and could bind directly to cysteine on carboxylate group of the ligand, which results in reducing the protein levels of γ-GCS and GSH-Px [40]. In addition, Cadmium could displace metal ions of intracellular metal-dependent enzymes, such as Se in GSH-Px via competitive or non-competitive alternatives. Moreover, after addition of SFN at different concentrations, the mRNA and protein expression of Nrf2 was increased in each group. Under the action of SFN, the mRNA and protein expressions of the downstream molecules, such as *HO-1*, *NQO1*, *γ-GCS,* and *GSH-Px* of the *Nrf2* signaling pathway increased in varying degrees. The above results indicated that SFN could activate the Nrf2 in TM4 cells and enhance the expressions of *HO-1, NQO1, γ-GCS*, and *GSH-Px*, thereby inhibiting oxidative stress, maintaining redox equilibria, reducing the oxidative damage of TM4 cells against Cd, and reducing the Cd-induced apoptosis.

In conclusion, this study confirmed that Cd caused the oxidative damage and apoptosis of TM4 cells, and SFN weakened the Cd-induced cell injury by activating the Nrf2/ARE signaling pathway. Therefore, these results provided a theoretical basis for the prevention and treatment of Cd-induced reproductive toxicity by SFN.

## Figures and Tables

**Table 1:** Primer sequence.

Gene	Accession No.	Primer Sequence (5′–3′)	Product Length
*Nrf2*	NM_010902.3	Forward: TCCTATGCGTGAATCCCAAT	103 bp
		Reverse: GCGGCTTGAATGTTTGTCTT	
*GSH-Px*	X03920.1	Forward: GAAGTGCGAAGTGAATGG	224 bp
		Reverse: TGTCGATGGTACGAAAGC	
*HO-1*	NM_010442.2	Forward: GGGCTGTGAACTCTGTCCAAT	162 bp
		Reverse: GGTGAGGGAACTGTGTCAGG	
*γ-GCS*	U85414.1	Forward: TGGATGATGCCAACGAGTC	185 bp
		Reverse: CCTAGTGAGCAGTACCACGAATA	
*NQO1*	NM_008706.5	Forward: TTCTGTGGCTTCCAGGTCTT	104 bp
		Reverse: TCCAGACGTTTCTTCCATCC	
*β-actin*	BC138614.1	Forward: CTGTCCCTGTATGCCTCTG	221 bp
		Reverse: TTGATGTCACGCACGATT	

**Table 2:** Effect of SFN on the apoptosis of Cd-induced TM4 cells.

Group	Apoptosis Rate (%)
Control (0 µmo/L)	4.3 ± 0.37
CdCl_2_ (12.5 µmo/L)	6.17 ± 0.82 *
SFN (0.625 µmo/L)	2.67 ± 0.62 **
SFN (2.5 µmo/L)	1.95 ± 0.14 **
SFN (10 µmo/L)	2.37 ± 0.08 **
CdCl_2_ (12.5 µmo/L) + SFN0.625 µmo/L	2.92 ± 0.18 ^##^
CdCl_2_ (12.5 µmo/L) + SFN2.5 µmo/L	2.52 ± 0.25 ^##^
CdCl_2_ (12.5 µmo/L) + SFN10 µmo/L	2.70 ± 0.35 ^##^

* *p* < 0.05 as compared to the control group,** *p* < 0.01 as compared to the control group. ^##^ *p* < 0.01 as compared to the Cd group.
